# Book Reviews

**Published:** 1913-03

**Authors:** 


### BOOK REVIEWS

### A NEW WORK ON THE HISTORY OF MEDICINE

W. B. Saunders Company, publishers, of Philadelphia and London, have in active preparation a work on the History of Medicine by Dr. Fielding H. Garrison, Principal Assistant Librarian, Surgeon-General's Office, and Editor of the Index Medicus. Dr. Garrison's twenty years' experience in medical bibliography, and the unusual advantages derived from his close touch with the rich stores of the Surgeon-General's Office, fit him most admirably for such a work as this.

His book will present the history of medicine from the earliest ancient and primitive times; on through Egyptian medicine, Sumerian and Oriental Medicine, Greek Medicine, the Byzantine Period; the Mohammedan and Jewish Periods, the Mediaeval Period, the Period of the Renaissance, the Revival of Learning and the Reformation; the Seventeenth Century (The Age of Individual Scientific Endeavor,) The Eighteenth Century (The Age of Theories and Systems,) the Nineteenth Century (The beginning of Organized Advancement of Science.) The Twentieth Century (the Beginning of Organized Preventive Medicine). There will also be Appendices covering Medical Chronology. Histories of Important Diseases, Histories of Drugs and Therapeutic Procedures, Histories of important Surgical Operations, and Bibliographic Notes for Collateral Reading.

Dr. Garrison's work will undoubtedly be a valuable book to every medical man. In this one volume he will get a complete history of medicine from its earliest times, presented in a concise form.

The illustrations are intended to stimulate the reader's interest in the picturesque aspects of medicine and in the personalities of its great leaders. The biographies will be confined to the most important facts and to interesting personal traits. The original bibliographic preferences to the important discoveries, operations and experiments will be given. Each period is to be followed by a brief survey of its social and cultural phases. Altogether it promises to be a most important addition to medical literature. We await its publication with much interest.

---

CHLORIDE OF LIME IN SANITATION. By Albert H. Hooker, Technical Director Hooker Electrochemical Co., John Wiley & Sons, New York.

This book comprises the data collected by the research department of the Hooker Electromechanical Co. regarding the use of chloride of lime in sanitation.

The work clearly showed that this inexpensive chemical was one of the most valueable and economical agents available for the protection, in many way, of the public health.

---

PROGRESSIVE MEDICINE. A quarterly digest of advances discoveries and improvements in the medical and surgical sciences. Edited by Hobart Amory Hare, M. D., Professor of Theradpeutics and Diagnosis in the Jefferson Medical College of Philadelphia. Assisted by L. F. Appleman, M. D., Instructor in Therapeutics, Jefferson Medical College. Vol. IV, December 1912. Lea & Febiger, Philadelphia.

E. H. Goodman gives an interesting review of the recent work upon the digestive tract and allied organs, the liver, pancreas and peritoneum, John R. Bradford discusses renal affections and begins with hemoglobemuria. Under the subject of nephritis of syphilitic origin he records three cases occurring during the course of secondary syphilis which causes death. They call attention to the importnce of recognizing this variety of nephritis which frequently escapes notice or its real nature is not recognized.

Genito-urinary diseases are reviewed by C. W. Bonny in a very instructive manner as are surgery of the extermities, chock anesthesia, infections, fractures and dislocations, and tumors, by J. C. Bloodgood. Practical therapeutics are summarized by H. R. Landis.

---

MEDICAL MEN AND THE LAW. A Modern Treatise on the Legal Rights, duties and Liabilities of Physicians and Surgeons. By Hugh Emmett Culbertson, of Ohio and New York Bars, Etc. Pp 325. Lea & Febiger, Philadelphia.

Culbertson deals ina ver yattractive and clear manner with the general principles and main features of modern law pertaining to physicians and surgeons. By careful selection he has given all that is substantial present importance with this volume of moderate size. The type is unusually large and clear and thus the reading easy.

---

Infections of the upper lip demand prompt and thorough treatment to avoid cerebral infections. Thrombosis of the nasolabial branch of the facial vein should be watched for. It appears as a redened cord which can be felt in the groove between the cheek and nose. Serious complications may be averted by excision of the thrombosed vein.—*American Journal of Surgery.*

